# Preparation, Characterization, and Keratinocyte Cell Viability of a β‑Cyclodextrin–Myrcene Complex Intended for Skin Application

**DOI:** 10.1021/acsomega.6c01078

**Published:** 2026-06-15

**Authors:** Felipe Mota Tashiro, Jonatas Lobato Duarte, Miquel Martínez-Navarrete, Antonio José Guillot, Ana Melero, Marlus Chorilli

**Affiliations:** † Department of Drugs and Medicines, School of Pharmaceutical Sciences of Araraquara, São Paulo State University, 01049-010 São Paulo, Brazil; ‡ Department of Pharmacy and Pharmaceutical Technology and Parasitology, 16781 University of Valencia, 46010 Valencia, Spain

## Abstract

*Context:* Myrcene has biomedical potential; however, due to its environmental instability and low water solubility, novel approaches, such as inclusion complex formation, can overcome these disadvantages and improve its applicability in cosmetics. *Objective:* This research investigated the formation of a β-cyclodextrin:myrcene complex using an experimental design approach, followed by physicochemical characterization. The inclusion complex was also evaluated for cytocompatibility in HaCaT keratinocytes for use in skin formulations. *Materials and methods:* Inclusion complexes were obtained by coprecipitation and optimized using response surface design. The complexes were characterized by FTIR spectroscopy, thermal behavior (TGA and DSC), XRD, and SEM. Cell viability in HaCaT cells was assessed using the MTT assay. *Results:* The β-cyclodextrin:myrcene complex exhibited a 75.88% inclusion efficacy. FTIR results indicated changes in the band position/intensity consistent with host–guest interactions. Thermal analyses revealed reduced water loss events and the disappearance of the β-CD thermal transition. The X-ray diffraction results suggest a reduction in crystallinity, as indicated by changes in the sample peaks. Scanning electron microscopy data reveal the formation of cubic/rhomboid structures. Cytocompatibility assays demonstrated that free myrcene maintained viability above 70% across most tested concentrations, whereas the formed inclusion complex reduced apparent viability at higher concentrations, consistent with limited aqueous solubility. *Conclusions:* β-CD effectively entrapped myrcene through coprecipitation, as confirmed by complementary solid-state and thermal analyses. Cytocompatibility results highlighted a reduction in cell viability after the formation of inclusion complexes when compared to myrcene and β-CD controls, possibly due to solubility-related effects. These findings reveal the need for the application of new methods to evaluate the *in vitro* compatibility and safety of intended skin use.

## Graphical abstract

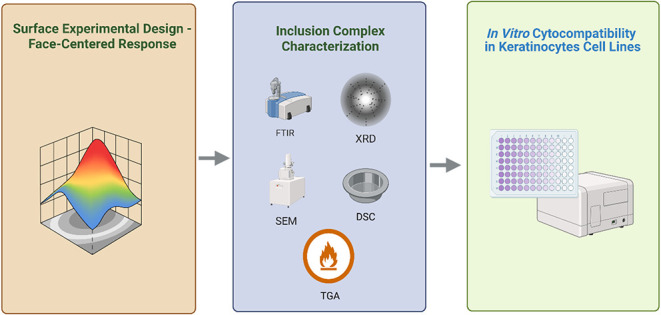

## Introduction

Terpenes are a large and diverse class of natural hydrocarbons that are biosynthesized by plants and insects from isoprene units. Depending on the number of isoprene moieties, they are classified as mono-, sesqui-, di-, or higher terpenes and are widely employed in food, cosmetics, pharmaceuticals, and material science applications. In addition to their industrial relevance as flavoring and fragrance agents, several terpenes exhibit bioactive properties, such as antioxidant, anti-inflammatory, antimicrobial, and anticancer effects, which have motivated their investigation as functional ingredients and biobased materials.^1,2^

Among monoterpenes, myrcene is a prominent acyclic compound found in the essential oils of several plant species, including bay, cannabis, hop, verbena, and cypress. Commercially available as β-myrcene (Figure 1c), it is an oily, hydrophobic molecule, characterized by three carbon–carbon double bonds, low molecular weight (136.2 g mol^–1^), and high volatility.^3,4^

**Figure 1:**
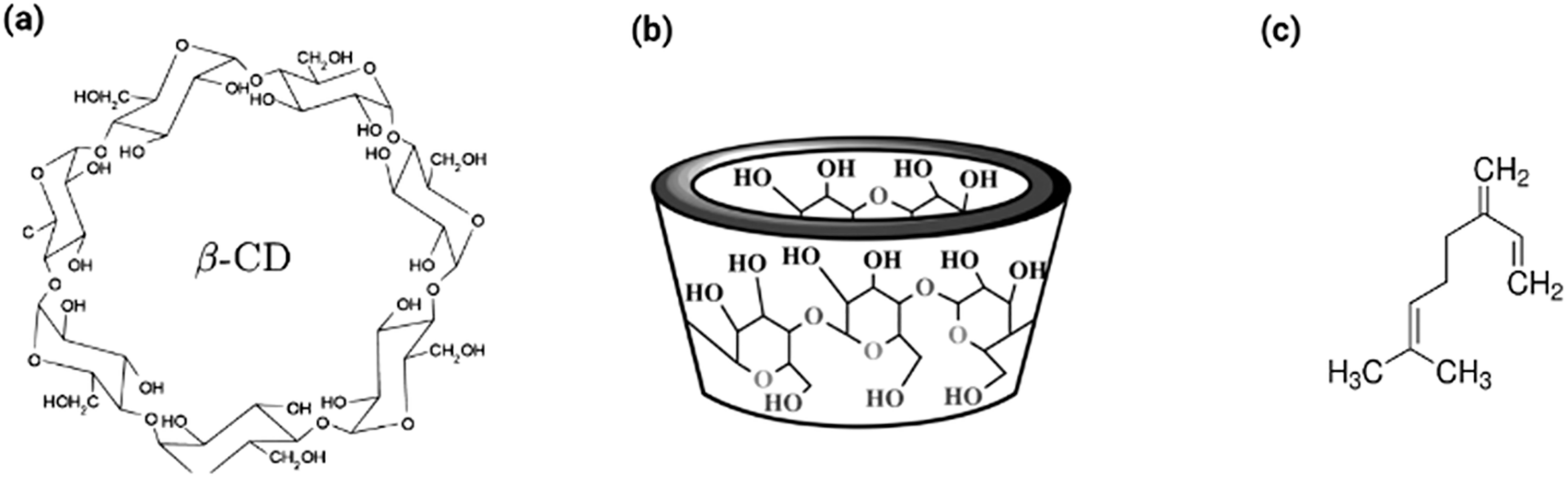
Structure of β-cyclodextrin (a), cone-shaped three-dimensional structure (b), and β-myrcene (c). Source: *Created in BioRender. Duarte, J. (2026). Agreement number: BV29NRBRBQ.* https://BioRender.com/wybqjfp.

Myrcene is used as a precursor for the synthesis of fragrances, polymeric materials, and elastomers, and has also attracted interest due to its reported biological activities.^1,4,5^ Myrcene possesses anti-inflammatory, antimicrobial, antioxidant, sedative, and insecticidal/repellent properties, demonstrating its potential to decrease oxidative stress, combat microbial infections, exert cytotoxic effects against certain cancer cells, and reduce inflammation.^6^

Despite their potential, most essential oils face a challenging problem regarding their usage due to their volatility, hydrophobic nature, sensitivity to light/oxygen, and heat. This environmental instability demands different approaches to improve its effectiveness and has attracted interest in the development of new systems. Nano- and microencapsulation are promising controlled alternatives, as they can achieve controlled release, physical instability, or even improve the safety of formulations. Also, complexation with natural and chemically modified cyclodextrins has been found to be an interesting strategy.^7,8^

β-cyclodextrin (β-CD) (Figure 1a,b) is obtained from starch degradation and is widely used as a carrier for the inclusion of lipophilic active compounds (with molecular weight <800 g mol^–1^), promoting dispersion on hydrophilic vehicles. Due to its hydrophilic exterior and hydrophobic cavity, it enables interaction with molecules such as myrcene through different interactions such as hydrophobic interactions, van der Waals forces, and hydrogen bonding, thereby promoting controlled drug release, enhancing dissolution rate, and reducing cytotoxicity. β-CD is the most extensively studied due to its benefits and cost despite its limited aqueous solubility. Inclusion complex systems are prepared using several methods, including physical mixing, kneading, atomization, freeze drying, spray drying, and coprecipitation, which rely on the intrinsic properties of the host and guest compounds.^9–16^

The present study focuses on developing and optimizing β-cyclodextrin–myrcene inclusion complexes using the coprecipitation method. The influence of process variables on powder recovery and inclusion efficiency was evaluated through experimental design. In addition, a cell viability assay was performed in HaCaT human keratinocytes, as a representative cell in skin tissues, to evaluate cellular responses and understand their safety and future applicability in topical formulations.

## Results and Discussion

### Analytical Method for Quantifying Myrcene

The validation of the UV–vis spectrophotometric method for myrcene quantification began with the acquisition of the absorption spectra in the 200–500 nm range, which revealed a maximum absorbance at 228 nm, selected for all subsequent quantitative analyses (Figure 2).

**Figure 2:**
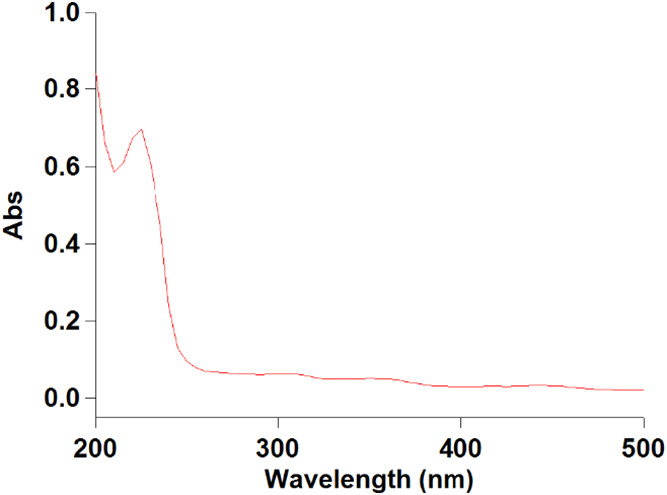
UV–vis scan spectra of β-myrcene in the 200–500 nm range.

Linearity was evaluated using independent analytical curves in a concentration range of 2.0–12 μg/mL. These curves demonstrated a strong linear relationship between absorbance and myrcene concentration, with correlation coefficients (*r* = 0.9998) and determination coefficients (*r* ^2^ = 0.9996) (Figure 3). Statistical analysis performed using one-way ANOVA confirmed the significance of the regression model, as evidenced by an F_calculated value (10,677.82) that was markedly higher than the F_tabulated value (7.71) and *p*-value <0.001 (Table 1), demonstrating a statistically significant linear fit within the evaluated range.

**Figure 3:**
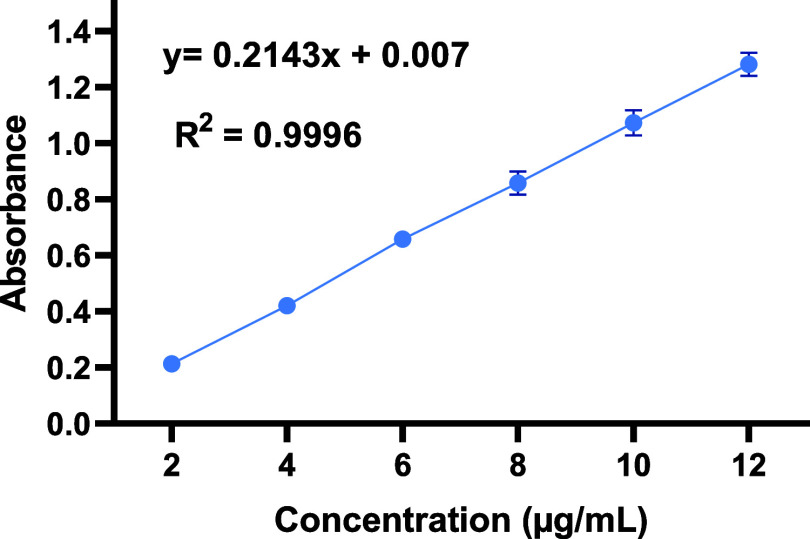
Analytical curve obtained by evaluating the linearity of β-myrcene (*n* = 3).

**Table 1:** Regression and ANOVA Analysis of Linearity

**Regression Statistics**	
correlation coefficient (*r*)	0.9998
coefficient of determination (*r* ^2^)	0.9996

**ANOVA**	
*F*_calculated	10,677.82
*F*_tabulated	7.71
*p*-value	<0.001

The method’s sensitivity was assessed by determining the limits of detection (LOD) and quantification (LOQ), which correspond to the analyte concentrations that can be accurately detected and quantified with adequate precision and accuracy, respectively. The LOD and LOQ values obtained for β-myrcene were 0.797 and 2.655 μg/mL, respectively, indicating adequate sensitivity for the quantification of myrcene in the inclusion complex structure.

Precision was determined by repeatability (intraday) and intermediate precision (interday) using the relative standard deviation (RSD) as the acceptance criterion. As presented in Table 2, the RSD values for all tested concentrations remained below 5% for both intra- and interday parameters, confirming the good precision and reproducibility of the analytical method.

**Table 2:** Precision Results at Different Concentrations (n = 3)

	**relative standard deviation (RSD)**	
**concentration** (μg/mL)	intraday	interday
**2**	3.748	1.363
**6**	3.706	3.374
**12**	3.488	2.972

The method’s accuracy was determined using the recovery test. The myrcene solution was added to the formulation matrix to assess three concentrations (Table 3). The recovery percentages ranged from 99.00% to 102.19% across all tested concentrations, with RSD values below 5%.

**Table 3:** Accuracy Results at Different Concentrations (*n* = 3)

**concentration** (μg/mL)	**experimental concentration** **(mg/mL)**	**recovery (%)**	**relative standard deviation (RSD)**
**2**	1.980	99.00	1.01
**6**	6.131	102.19	2.14
**12**	11.951	99.60	0.40

The robustness of the analytical method was assessed by modifying two parameters independently: the monoterpene solvent and the spectrophotometer equipment used. The results of this robustness analysis are summarized in Table 4.

**Table 4:** Effect on Modification Conditions on the Quantification of Myrcene at 6 μg/mL (*n* = 3)

**variable**	**experimental concentration** **(mg/mL)**	**recovery (%)**	**relative standard deviation (RSD)**
**solvent (ethanol)**	6.384	106,40	6,01
**equipment (UV-1800; Shimadzu, Kyoto, Japan)**	5.534	92.24	8.42

The results presented above indicate that variations in both variables had a moderate impact on myrcene quantification, as indicated by the recovery percentages and relative standard deviations (RSD). The results demonstrate the method’s capacity to deliver reliable and accurate results despite variations in solvents and equipment.

### Optimization of Inclusion Complexes through Experimental Design

The β-cyclodextrin:myrcene inclusion complexes were prepared using a face-centered response surface experimental design to evaluate the influence of two independent variables: the myrcene:β-CD mass ratio (X_1_) and the ethanol:water volumetric ratio (X_2_). These parameters provided relevant insights into the ideal conditions for maximizing the formation of the inclusion complex.^17^

Response surface analysis was performed using a second-order polynomial regression model fitted by response surface regression, according to the central composite face-centered design. The results were assessed using the following regression equation (eq 1).

1 $$Y=\beta_{o}+\beta_{i}X_{i}+\beta_{j}X_{j}+\beta_{\mathrm{ii}}X_{i}^{2}+\beta_{\mathrm{jj}}X_{j}^{2}+\beta_{\mathrm{ij}}X_{i}X_{j}+E$$

Selected variables were coded, where *Y* refers to the predicted responses, β_0_ is the constant coefficient, β_*i*_, β_*ii*_, and β_*ij*_ are the coefficients of linear, quadratic, and interaction effects, respectively, *X*_*i*_ and *X*_*j*_ represent independent variables, and *E* is the error.

Two processing conditions were followed and compared (Figure 12): heating for 30 min after the addition of myrcene (Process 1) or immediate cooling followed by stirring at room temperature (Process 2). Powder recovery (%) and myrcene inclusion efficiency (%) were used as the response variables. The full experimental matrix and results are provided in the Supporting Information (Tables S1–S4).

For the response surface regression analysis in Process 1, powder recovery was influenced by the myrcene:β-CD ratio, in both linear and quadratic terms, showing statistical significance with a *p*-value <0.05. On the contrary, the ethanol:water ratio and the interaction between factors did not significantly affect this response. Interaction graphs (Figure 4a) indicate that the highest powder recovery values were primarily associated with intermediate myrcene loading (15:85).

**Figure 4:**
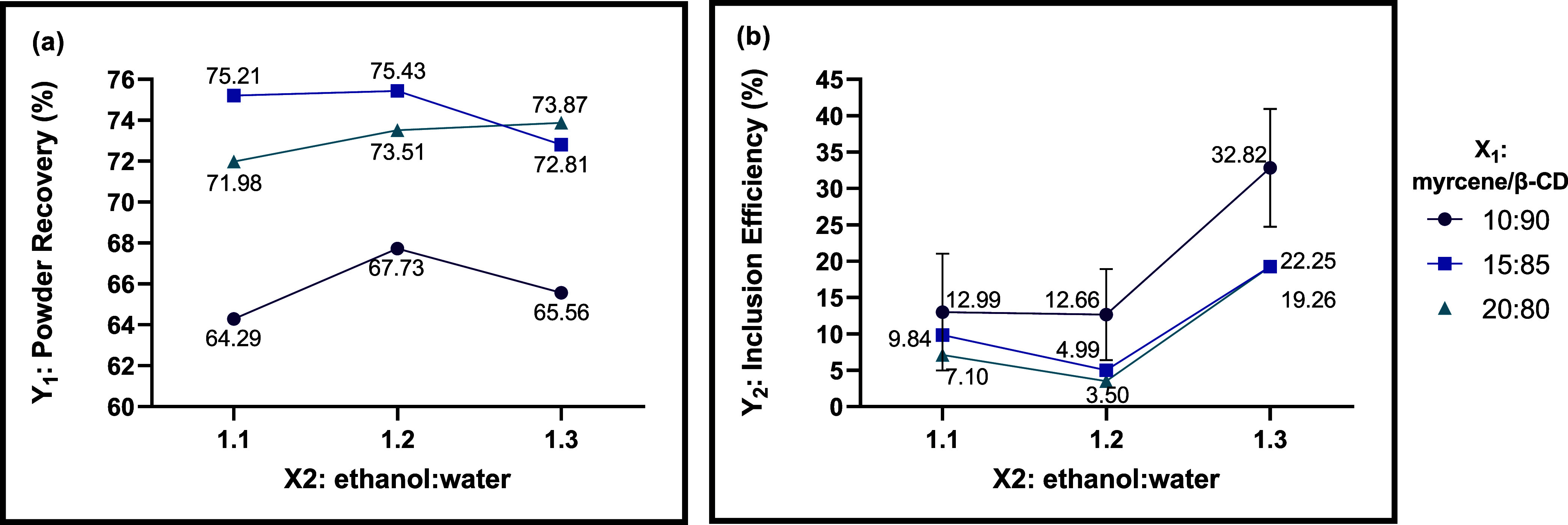
Interaction between independent factors for % powder recovery (a) and inclusion efficiency (b) in Process 1.

In Process 1, the Inclusion efficiency was affected by both independent variables. The negative coefficient associated with the myrcene:β-CD ratio indicates that increasing myrcene content affects the encapsulation efficiency, suggesting a saturation effect in the cyclodextrin cavity. Additionally, the ethanol:water ratio exhibited significant linear and quadratic effects, with a higher water content favoring the inclusion efficiency (Figure 4b). In addition, no statistically significant interaction between the factors was observed, indicating that their effects on encapsulation were independent.

Regression analysis for Process 2 showed no statistically significant linear, quadratic, or interaction effects for either powder recovery or inclusion efficiency at a *p*-value of <0.05, in contrast to the results obtained during Process 1, even though the interaction plots (Figure 5) displayed clear visual trends, with higher inclusion efficiencies consistently associated with higher water content in the ethanol:water mixture and lower myrcene loading ratios.

**Figure 5:**
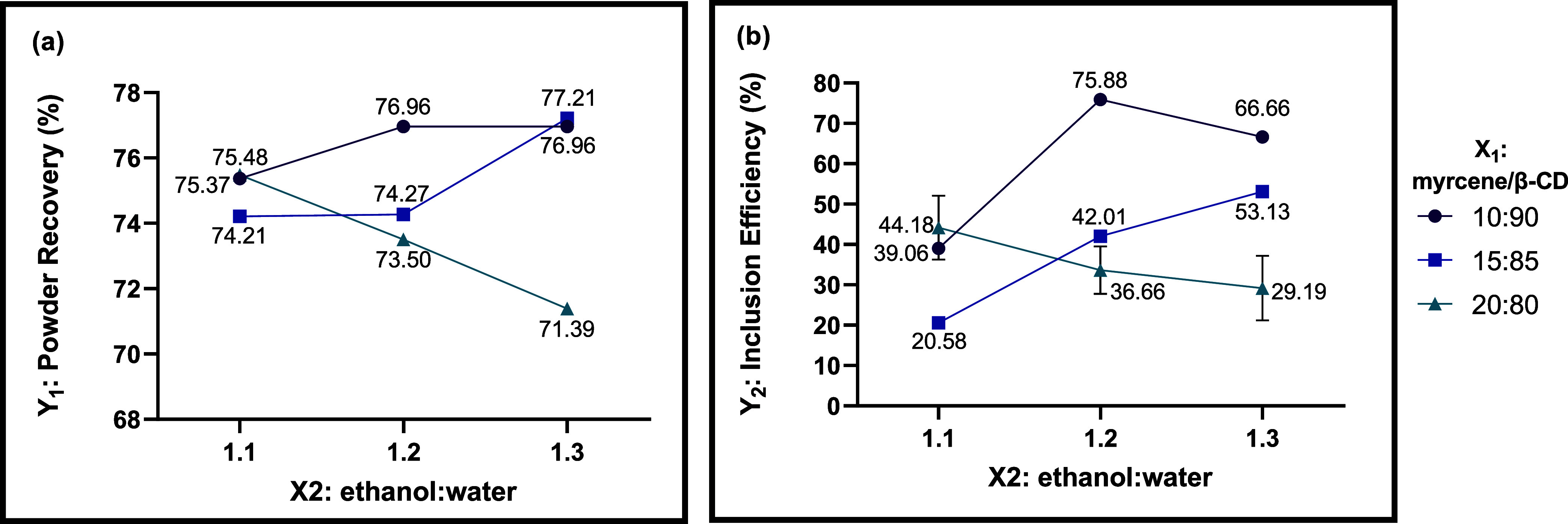
Interaction between independent factors for % powder recovery (a) and inclusion efficiency (b) in Process 2.

Moreover, in Process 2, substantially higher inclusion efficiency values were obtained compared with those obtained in Process 1, particularly at a myrcene:β-CD ratio of 10:90 combined with ethanol:water ratios of 1:2 and 1:3. These results suggest that avoiding prolonged heating minimizes myrcene volatilization and preserves its availability for host–guest complexation within the β-CD cavity.

As shown in Figure 5a, among the three highest initial mass recovery (Y1) values, we identified one point at a myrcene:β-CD ratio of 15:85 (77.21%) and two points at a 10:90 ratio (76.96 and 76.96%), and one at each ethanol:water ratio. On the other hand, in the graph presented in Figure 5b, the three highest inclusion efficiency values occur at an ethanol:water ratio of 1:3 but are distributed across the three myrcene:β-CD ratios of 10:90 (66.66%), 15:85 (53.13%), and 20:80 (29.19%).

The analysis of the graphs in Figures 4 and 5 demonstrates that both the myrcene:β-CD ratio and ethanol:water ratio have important effects on powder recovery and inclusion efficiency, increasing or decreasing their content. The highest powder recovery efficiency was observed at myrcene:β-CD ratios of 15:85 and 10:90, while the ethanol:water ratio of 1:3 was optimal for the inclusion efficiency, with the myrcene:β-CD ratio of 10:90 providing the best inclusion efficiency results in this context.

The coprecipitation method is used to complex water-insoluble substances, promoting their interaction with cyclodextrins through hydrophobic or van der Waals interactions when added to ethanolic solutions.^18^ However, using ethanol as a solvent for myrcene in excess prevents the interaction of the terpene with cyclodextrin. This is evident in the results with the 1:3 ratio, which shows better encapsulation efficiency in both processes and powder recovery in process 2.^19^

Regarding the differences between the results of the production processes of the inclusion complexes, it is possible to verify, through the *t-*test application for 2 samples, the differences in the factors dependent on powder recovery (%) and inclusion efficiency (%) (Table 5).

**Table 5:** T-Test Results with Descriptive Statistics and Confidence Intervals for Comparison between Processes 1 and 2^a^

	**descriptive statistics**				**estimate of the difference**		***t-*test**
**dependent Factors**	***N***	**average**	**std**	**mean std**	**difference**	**95% CI**	***p*-value**
**% recovery of P1 powder**	9	71.15	4.2	1.4	–3.88	(−7.27; −0.50)	0.028
**% recovery of P2 powder**	9	75.04	1.92	0.64			
**inclusion efficiency P1**	9	13.93	9.42	3.1	–31	(−45.56; −16.43)	0.001
**inclusion efficiency P2**	9	44.9	17.7	5.9			

a Abbreviations: **CI**, confidence interval; **P1**, process 1; **P2**, process 2; **Std**, standard deviation; **N**, number of samples.

The inclusion complexes obtained by process 1 showed 71.15 ± 4.2% powder recovery and 13.93 ± 9.42% inclusion efficiency for the monoterpene, compared to 75.04 ± 1.92% powder recovery and 44.93 ± 17.7% inclusion efficiency for process 2. The difference in these processes may be attributed to solvent volatilization or even to the alteration of myrcene characteristics during heating at 55 °C, affecting the solubilization and/or concentration of the compound in the complexation system.^20^ With difference estimates of −3.88 and −31 and 95% confidence intervals for the difference different from zero, it reinforces that there is a significant difference among the means, as the interval does not include zero.

A comparison between the two processing conditions confirmed a statistically significant difference in both powder recovery and inclusion efficiency. The p-values obtained (0.0028 for powder recovery and 0.001 for inclusion efficiency) were below the established significance threshold (<0.05), reinforcing that the application of postaddition heating significantly affects the production of the β-cyclodextrin–myrcene complex system.

The myrcene:β-cyclodextrin ratio (X_1_) in the 15:85 inclusion complex consistently yielded powder recovery values exceeding 75% under both processing conditions. However, the inclusion efficiency at this ratio varied markedly across processes, ranging from 32.82% in Process 1 to 75.88% in Process 2. This behavior reflects the finite loading capacity of the β-cyclodextrin cavity, in which excess myrcene remains noncomplexed and is subsequently removed during postcrystallization washing or lost through volatilization, particularly under heating conditions (Process 1).^21^

On the basis of these findings, three inclusion complexes obtained under Process 2 were selected for further physicochemical characterization: 10:90 (ethanol:water 1:2), 10:90 (1:3), and 15:85 (1:3), which exhibited inclusion efficiencies of 75.88, 66.66, and 53.13%, respectively.

### Characterization of the Inclusion Complex

#### Fourier Transform Infrared (FTIR) Spectroscopy

The spectra of myrcene, β-CD, and physical mixtures at ratios of 10:90 and 15:85 were used as controls for the samples selected after the design process (10:90 1:2, 10:90, 1:3, and 15:85 1:3).

The characteristic bands belonging to β-CD (Figure 6a) are observed, which display a broad band at 3396 cm^–1^, corresponding to the stretching vibrations of the −OH groups and a band at 2930 cm^–1^ for the CH bonds. The region at 1631.5 cm^–1^ is attributed to the OH bending vibration of adsorbed water molecules, while the broad band at 1421 cm^–1^ corresponds to the overlap between CH_2_ and OH in the planar bending bands. It also presents intense bands at 1037 and at 1174.7 cm^–1^, which can be attributed to the stretching vibrations of the CO, CC, and CO–OC bonds.^12,21–23^

**Figure 6:**
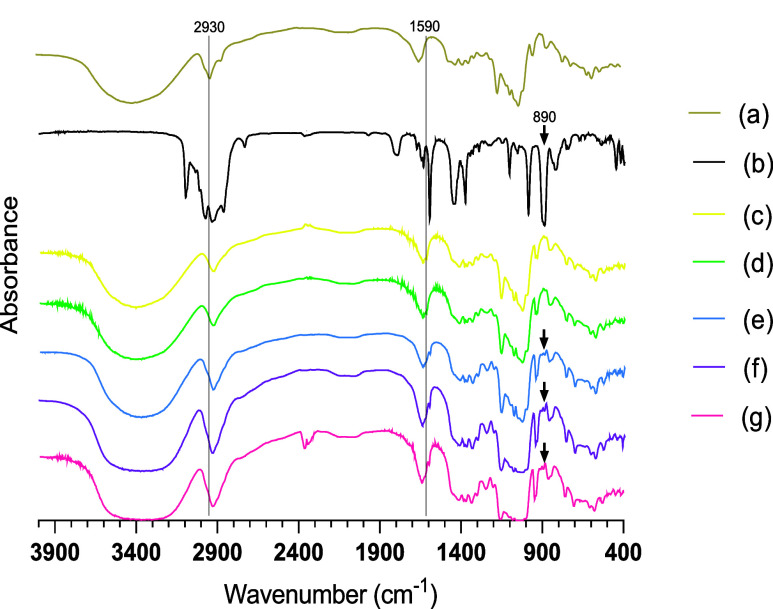
FTIR spectra in the 500–4000 nm range. **Legend:** (a) β-CD, (b) myrcene, (c) physical mixture 10:90, (d) physical mixture 15:85, (e) sample 10:90 1:2, (f) sample 10:90 1:3, and (g) sample 15:85 1:3.

Myrcene (Figure 6b) exhibits a characteristic band at 3094 cm^–1^, attributed to double bonds. The region between 2971–2859.9 cm^–1^ is associated with stretching vibrations of the methyl and alkane groups. The signals observed between 1645 and 1608 cm^–1^ correspond to the C=C bonds. The absorbance at the 1380 cm^–1^ region is attributed to the deformation vibrations of the CH_2_ radicals, while the two bands at 886.3 and 898.4 cm^–1^ are characteristics of the =CH terminal bonds.^24–26^

The physical mixtures in the proportions of 10:90 and 15:85 (Figure 6c,d) showed great similarity to each other, presenting the main absorption bands characteristic of both β-CD and myrcene. Among the variations observed, the region between 2971 and 2859.9 cm^–1^ stands out, corresponding to the methyl groups of myrcene, whose absorption partially overlapped with the intense signal of β-CD.^22^ Furthermore, a striking similarity is observed at around 886.3 cm^–1^, where the band intensity increased in the physical mixtures compared to that in free β-CD, which can be attributed to the presence of =C–H bonds originating from the unsaturated structure of myrcene.^26^

The FTIR spectra obtained from the inclusion complexes 10:90 1:2 (e), 10:90 1:3 (f), and 15:85 1:3 (g) showed no significant variations among them. However, some bands differ between the control and the inclusion complex spectra. An example is the band corresponding to the CH bonds observed at 2930 cm^–1^ for β-CD, which shifts slightly to 2900 cm^–1^ across all samples. Although this shift is subtle, variations in band intensity are observed between the physical mixture controls and the inclusion complexes, indicating alterations in the chemical microenvironment of the C–H groups. Furthermore, since myrcene also exhibits absorption bands in the region between 2971 and 2859.9 cm^–1^, attributed to methyl and alkane groups, a higher absorption intensity is observed in the samples corresponding to the inclusion complexes, suggesting an overlap of the characteristic vibrations of myrcene and β-CD.

Another relevant difference is the emergence of a small band at 1590 cm^–1^, which is close to that of the C=O groups. In physical mixtures, this band is partially obscured by the intense absorption of β-CD, making it difficult to distinguish it. However, in the inclusion complexes, the band becomes well-defined, suggesting alterations in the intermolecular interactions or greater exposure of these vibrations due to the encapsulation of myrcene.

Finally, in the region around 890 cm^–1^, characteristic of the =CH terminal bonds of myrcene, the band is masked in the spectra of β-cyclodextrin and physical mixtures due to the overlapping of the host matrix bands, but in the inclusion complexes, this band is detected, showing the partial preservation of the myrcene characteristics, suggesting that, although included, the compound maintains part of its structure exposed outside the β-CD cavity.^27,28^

#### Thermogravimetric Analysis (TGA)

Thermogravimetric analysis (TGA) was employed to understand the thermal stability and mass loss changes in the behavior of substances and particles as a function of temperature. This study provides insights into the physicochemical changes associated with host–guest interactions.^22^ TGA was applied to investigate the formation of cyclodextrin inclusion complexes through modifications by characteristic thermal events, such as dehydration and thermal degradation, reflecting structural rearrangements within the cyclodextrin cavity.^12,29^ The TGA thermograms are shown in Figure 7.

**Figure 7:**
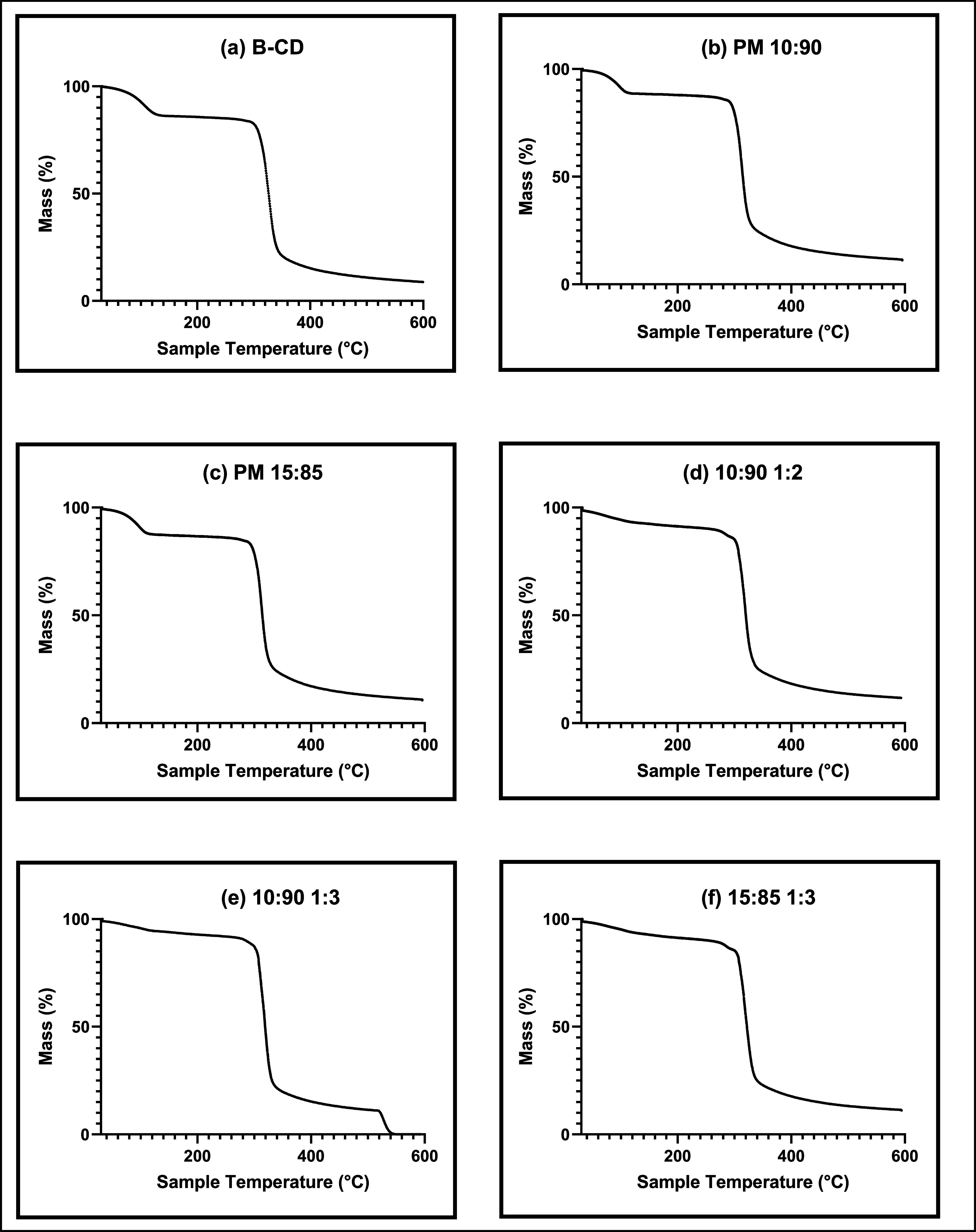
Thermograms related to TGA analysis. **Legend**: (a) β-CD (β-cyclodextrin), (b) PM 10:90 (physical mixture 10:90), (c) PM 15:85 (physical mixture 15:85), (d) 10:90 1:2 (sample 10:90 1:2), (e) 10:90 1:3 (sample 10:90 1:3), and (f) 15:85 1:3 (sample 15:85 1:3). Sample (e) 10:90 1:3 was interrupted during the analyses due to a software error, causing 0% mass at 520 °C.

The thermogram of β-cyclodextrin displayed multistage thermal mass loss events. The first event, at around 100 – 120 °C, corresponds to a 14% mass loss of adsorbed water molecules retained in the internal cavity of the structure. The second event, at around 300–350 °C, is associated with the thermal degradation of the β-CD matrix, corresponding to a 65% mass loss.^17,29^

Physical mixtures (Figure 7b,c) displayed mass loss profiles similar to those of pure β-cyclodextrin and the inclusion complexes. In particular, the first mass loss event in these controls was 10–12%. The second mass loss occurred in a similar temperature range, 300–350 °C, with a mass loss of 57–60%. A reduction in the initial mass loss was observed, which may be attributed to the partial displacement of cavity-associated water molecules by myrcene in the solid mixture, even in the absence of true inclusion complex formation.^29^

The thermograms (d), (e), and (f) show a significant reduction in mass loss in the temperature range of 100–120 °C, with approximately 5–8% of the initial mass lost in the formed inclusion complexes and 60–70% at the β-CD degradation temperature. The reduced dehydration at 100–120 °C indicates that the water previously present in the cyclodextrin cavity was replaced by myrcene molecules during the complexation process. Additionally, intermolecular interactions, such as hydrogen bonds between the host and inner cavity of the β-CD, contribute to the thermal stabilization of the complex and the new cyclodextrin degradation profile observed above 300 °C.^12,29,30^

#### Differential Scanning Calorimetry (DSC)

Differential scanning calorimetry (DSC) was employed to verify the thermal modifications associated with dehydration, crystalline rearrangement, and degradation processes, providing complementary information to thermogravimetric analysis. Cyclodextrin thermal behavior changes upon host–guest interactions; this assay demonstrates how inclusion complexation can modify dehydration enthalpy and crystalline phase transitions.^22,29^

The thermal profile of β-cyclodextrin (Figure 8a) presentes 3 endothermic events: the first one at around 100 °C, due to internal cavity dehydration; the second, represented by a small event at around 210 °C, refers to the crystalline phase transition of cyclodextrin; and the last, starting at around 250 °C, is attributed to the thermal degradation of the cyclodextrin structure, in agreement with previous reports.^29^

**Figure 8:**
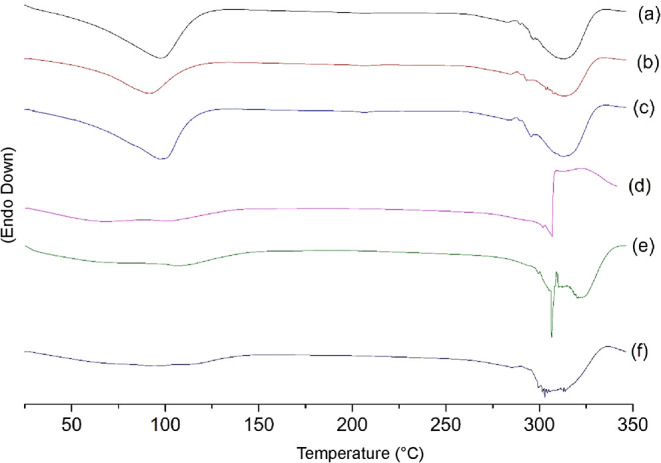
Thermograms related to DSC analysis. **Legend:** (a) β-CD (β-cyclodextrin), (b) PM 10:90 (physical mixture 10:90), (c) PM 15:85 (physical mixture 15:85), (d) 10:90 1:2 (sample 10:90 1:2), (e) 10:90 1:3 (sample 10:90 1:3), and (f) 15:85 1:3 (sample 15:85 1:3).

In the physical mixtures (Figure 8b,c), the same events were noticed as those in β-cyclodextrin, indicating the absence of major structural modifications. However, the physical mixture with a myrcene:β-CD ratio of 10:90 showed a lower enthalpy associated with the dehydration event, which may be attributed to a partial reduction in the amount of water retained within the cyclodextrin cavity due to superficial interactions between myrcene and the cyclodextrin.^30^

In the ICs (d), (e), and (f), the near occlusion of the water loss event and the dissipation of the peak at 210 °C provide convincing evidence of an inclusion complex between myrcene and β-CD. However, partial dehydration of the cyclodextrin cavity is also possible during the process. To obtain more conclusive evidence, complementary techniques are needed. Similar to TGA, the occlusion of this event indicates that water in the internal structure is replaced by myrcene, forming the interaction necessary to stabilize the terpene in the cyclodextrin cavity.^22,29,30^

#### X-ray Diffraction (XRD)

X-ray diffraction (XRD) was performed to evaluate the changes in the β-cyclodextrin crystal structure resulting from its interaction with myrcene. In its native crystalline state, β-cyclodextrin exhibits a highly ordered supramolecular structure stabilized by extensive intra- and intermolecular hydrogen bonding, producing well-defined diffractograms with sharp and intense peaks.^29^

β-Cyclodextrin (Figure 9a) exhibited diffraction peaks (2θ) at approximately 9, 12, 22, 27, and 35°, confirming its high crystallinity in the sample, in agreement with the results reported in the literature.^29,30^

**Figure 9:**
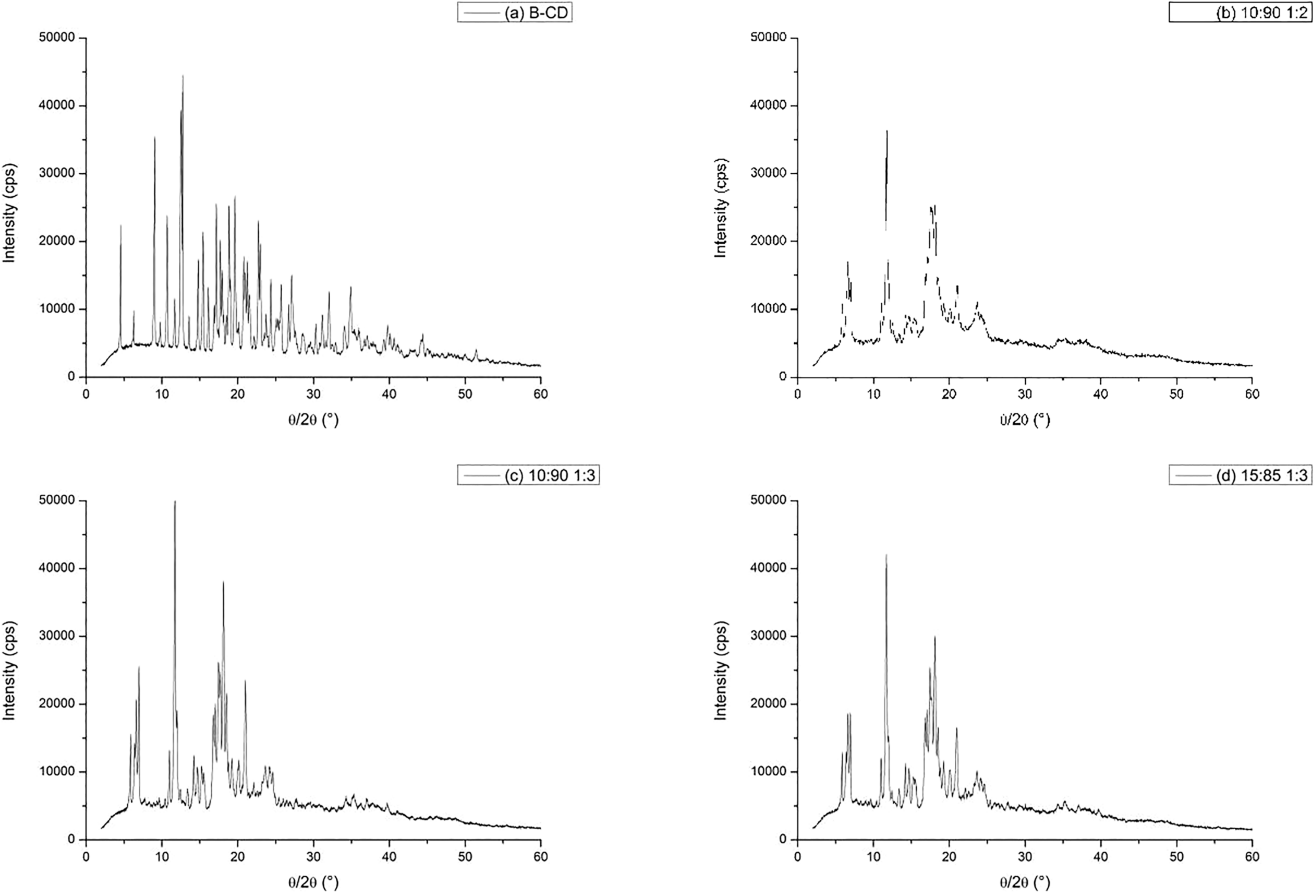
Diffractograms related to XRD analysis. **Legend:** (a) β-CD (β-cyclodextrin), (b) 10:90 1:2 (sample 10:90 1:2), (c) 10:90 1:3 (sample 10:90 1:3), and (d) 15:85 1:3 (sample 15:85 1:3).

After the formation of inclusion complexes, changes in the diffraction pattern of β-CD may include the disappearance, appearance, or displacement of the characteristic peaks.^22^ In the case of the developed ICs (b), (c), and (d), a reduction in crystallinity is observed with a reduction in the peaks in the samples. It is evident that, in all 3 samples analyzed, a new peak appeared around the 7° angle while the characteristic cyclodextrin peaks at 12 and 22° were retained. In general, the reduction in β-CD and the emergence of new diffraction peaks suggest the formation of a new supramolecular structure, indicating complexation between β-cyclodextrin and myrcene, as shown in Figure 9b,c,d.

#### Scanning Electron Microscopy (SEM)

The cyclodextrin surfaces and inclusion complexes were evaluated using scanning electron microscopy (SEM). This assay allows the analysis of morphological changes associated with the formation of β-cyclodextrin–myrcene inclusion complexes. This complementary technique is valuable for assessing the surface morphology and particle organization following physicochemical transformations, such as inclusion complexation.^12^

The cyclodextrin, β-cyclodextrin, presented in its native form, exhibited an irregular crystalline morphology, characterized by clusters of particles of varying sizes and a rough surface, as shown in Figure 10(a1) and (a2) at 500× and 1000× magnifications.^17^

**Figure 10:**
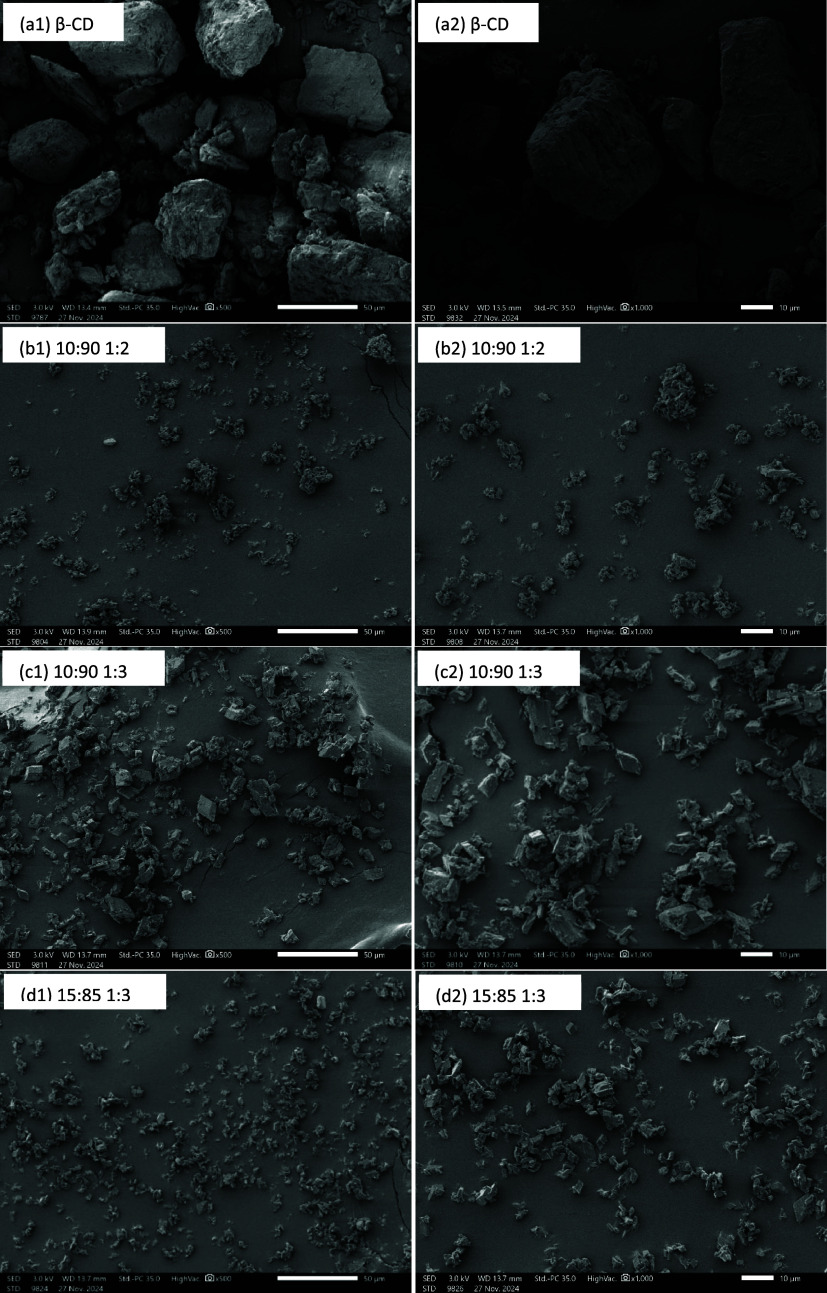
Scanning electron microscopy (SEM). **Legend:** (a1) β-CD (β-cyclodextrin) at 500x and (a2) at 1000x, (b1) 90 1:2 (sample 10:90 1:2) at 500x and (b2) at 1000x, (c1) 10:90 1:3 (sample 10:90 1:3) at 500x and (c2) at 1000x, and (d1) 15:85 1:3 (sample 15:85 1:3) at 500x and (d2) at 1000x.

The formation of inclusion complexes is suggested by a comparative analysis of micrographs, which shows two main morphological modifications: reduction in the particle size and the formation of more cuboid/rhomboid structures.^12,17^

Finally, SEM revealed a significant morphological change in the formed complexes when compared to pure β-cyclodextrin, analyzed at the same magnifications (500× and 1000×). A clear reduction in the particle size is observed, particularly in the 10:90 1:2 sample, which shows less agglomeration and more individualized particles. This reduction in particle size can be attributed to the strong intramolecular interactions formed between myrcene and β-CD during complexation, which promote the structural reorganization and stabilization of the new supramolecular form.^30^

The samples 10:90 1:3 (Figure 10 c2) and 15:85 1:3 (Figure 10 c2), at 1000× magnification, exhibit particles with a more regular morphology, closer to cubes and/or rectangular prisms, and a less rough surface compared to β-CD. These modifications indicate the formation of a more ordered and compact structure, characteristic of well-formed inclusion complexes, corroborating the results obtained from the XRD and TGA analyses.

### *In* Vitro Cytocompatibility in Keratinocyte Cell Lines

Keratinocyte viability was assessed by measuring the metabolic activity after cell exposure to different concentrations of β-cyclodextrin:myrcene inclusion complexes, raw β-CD, and myrcene. As the main constituent of the epidermal structure, the HaCaT cell line was used as a representative skin cell.^31^ Additionally, the myrcene concentrations tested were considered to represent 100% inclusion complexation, even though the inclusion complex sample had an inclusion efficiency of 75.88%.

Myrcene (Figure 11) shows cell viability above 70% across a concentration range of 426.17–13.31 μg/mL and, in some cases, above 100%. As described in the literature, this may be related to survival signaling through the ERK1/2 (MAPK) pathway or reduced oxidative stress that promotes mitochondrial activity.^3,32^

**Figure 11:**
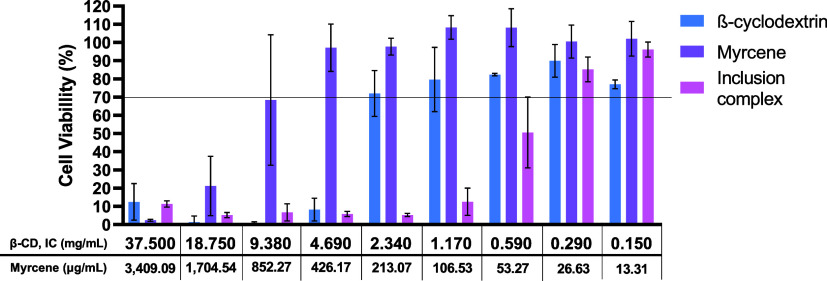
HaCaT cell viability through the MTT assay. Myrcene and β-cyclodextrin were used as controls, while the inclusion complex sample 10:90 1:2 was tested. Statistical results by one-way ANOVA showed significance of <0.05 (P-value 0.0475).

The β-cyclodextrin and inclusion complex results (Figure 11) show low cell viability compared to myrcene. The raw β-CD showed viability over 70% in the range of 2.340–0.150 mg/mL, while the β-cyclodextrin:myrcene complex showed viability over 70% at 0.290–0.150 mg/mL. These results are attributed to β-cyclodextrin’s low solubility in the medium, which forms a layer of particles over the cells, blocking CO_2_ from reaching the cells and causing cellular hypoxia, mitochondrial stress, and reduced NADPH-dependent activity, thereby reducing the formation of formazan crystals.^12,33–35^

These findings demonstrate an assessment of the cytocompatibility and concentration ranges of free myrcene, β-CD, and the inclusion complex, rather than as direct evidence that β-CD complexation improves the biological performance of myrcene; as well as support the selection of noncytotoxic concentrations for subsequent biological assays.

## Conclusions

The β-cyclodextrin:myrcene inclusion complex developed in this study exhibited high encapsulation efficiency, reaching 75.88% at a myrcene:β-CD ratio of 10:90 and an ethanol:water ratio of 1:2 under optimized conditions. Comprehensive physicochemical characterization using FTIR spectroscopy, thermal analysis, X-ray diffraction, and scanning electron microscopy revealed consistent structural, thermal, and morphological modifications of the β-cyclodextrin matrix, supporting the formation of host–guest interactions between myrcene and the cyclodextrin cavity.

Cytocompatibility assessment in HaCaT keratinocytes indicated that free myrcene maintained high cell viability across most tested concentrations, whereas β-cyclodextrin and the inclusion complex showed reduced apparent viability at higher concentrations. This effect may be associated with the solubility and concentration-dependent limitations of cyclodextrin systems rather than the intrinsic cytotoxicity of the inclusion complex itself. These results highlight an important preliminary step of defining appropriate concentration ranges when evaluating cyclodextrin inclusion complexes in cell-based assays.

Overall, the results indicate that β-cyclodextrin is an effective carrier for myrcene inclusion and a promising strategy for improving the physicochemical handling and potential stabilization of this monoterpene. The obtained IC structure provides a promising foundation for developing topical and controlled-release formulations, in which the formulation parameters can be tailored to balance the encapsulation efficiency and biological compatibility.

## Methods

### Materials

β-Myrcene (>98.0%), β-Cyclodextrin (≥97.0), and 3-(4,5-dimethylth-iazol-2-yl)-2,5-diphenyltetrazolium bromide (MTT) were purchased from Sigma-Aldrich (St. Louis, USA). Ethanol, acetonitrile, and HPLC-grade water were obtained from Merck (Darmstadt, Germany).

### Analytical Method for Quantifying Myrcene

A UV–Vis spectrophotometric method was employed for myrcene quantification. Initially, a UV absorption scan of a myrcene solution (5 μg/mL in ethanol) was recorded in the range of 200–500 nm to establish the wavelength of the highest molar absorptivity using a UV–vis spectrophotometer (Shimadzu, Kyoto, Japan).

Analytical method validation was performed according to the ICH Guidelines on the Validation of Analytical Procedures and the FDA Guidance for Bioanalytical Methods to determine the parameters of linearity, precision, accuracy, limit of detection, limit of quantification, and robustness.^36–38^

(a) Linearity. For linearity, three independent analytical curves were analyzed, each with six myrcene concentrations in the range of 2.0–12 μg/mL. The results were evaluated by visual analysis of the response as a function of analyte concentration, regression statistics (determination and correlation coefficients), equation of the line determined by the ordinary least-squares method, and evaluation of the significance of the slope and y-intercept. Statistical analysis was performed using one-way ANOVA with α=95%.
(b) Limit of detection (LOD) and limit of quantification (LOQ). These parameters were determined using the average values of the analytical curves (*n* = 3) for which the standard deviation of the response and the slope of the curve were obtained using the linear regression model, according to eqs 2 and 3:
2 LOD=3.3(σ/S)
3 LOQ=10(σ/S)
  where σ is the standard deviation of the response, and *S* is the slope of the calibration curve.
(c) Precision. The precision was determined by repeatability (intraday) and intermediate precision (interday). Intraday repeatability was measured using three myrcene solutions (2, 6, and 12 μg/mL) on the same day under the same experimental conditions. The intermediate precision (interday) of the method was evaluated by performing the analysis on two different days (interday). Intraday and interday precisions were expressed as relative standard deviations (RSDs).
(d) Accuracy. The accuracy was determined by comparing the theoretical result with the result obtained using three concentrations (2, 6, and 12 μg/mL) of myrcene used in the formulation matrix. Accuracy was evaluated using the coefficient of variation and the percentage of recovery.
(e) Robustness. Robustness of the method was determined by independently varying two parameters: the solvent, in which myrcene was dissolved in acetonitrile instead of ethanol, and the spectrophotometer model, which was changed to UV-1800 (Shimadzu, Kyoto, Japan). For this parameter, the theoretical result was compared with the result obtained at 6 μg/mL myrcene. The coefficient of variation and percentage of analyte recovery were used to evaluate accuracy.

### Obtaining Myrcene Inclusion Complexes in β-Cyclodextrin and Optimization

#### Preparation of Inclusion Complexes Complexes Using Different Proportions of β-Cyclodextrin:Myrcene

Myrcene:β-CD complexes were obtained using the coprecipitation method (Figure 12). 1 g of β-CD was dispersed in 10 mL of a hydroalcoholic solution in the following proportions (ethanol:water): 1:1, 1:2, and 1:3 (v:v). Concomitantly, a 10% ethanolic solution of myrcene was prepared and added to the previous solution in the following proportions of myrcene:β-cyclodextrin (w:w): 10:90 (10%), 15:85 (15%), and 20:80 (20%).

**Figure 12:**
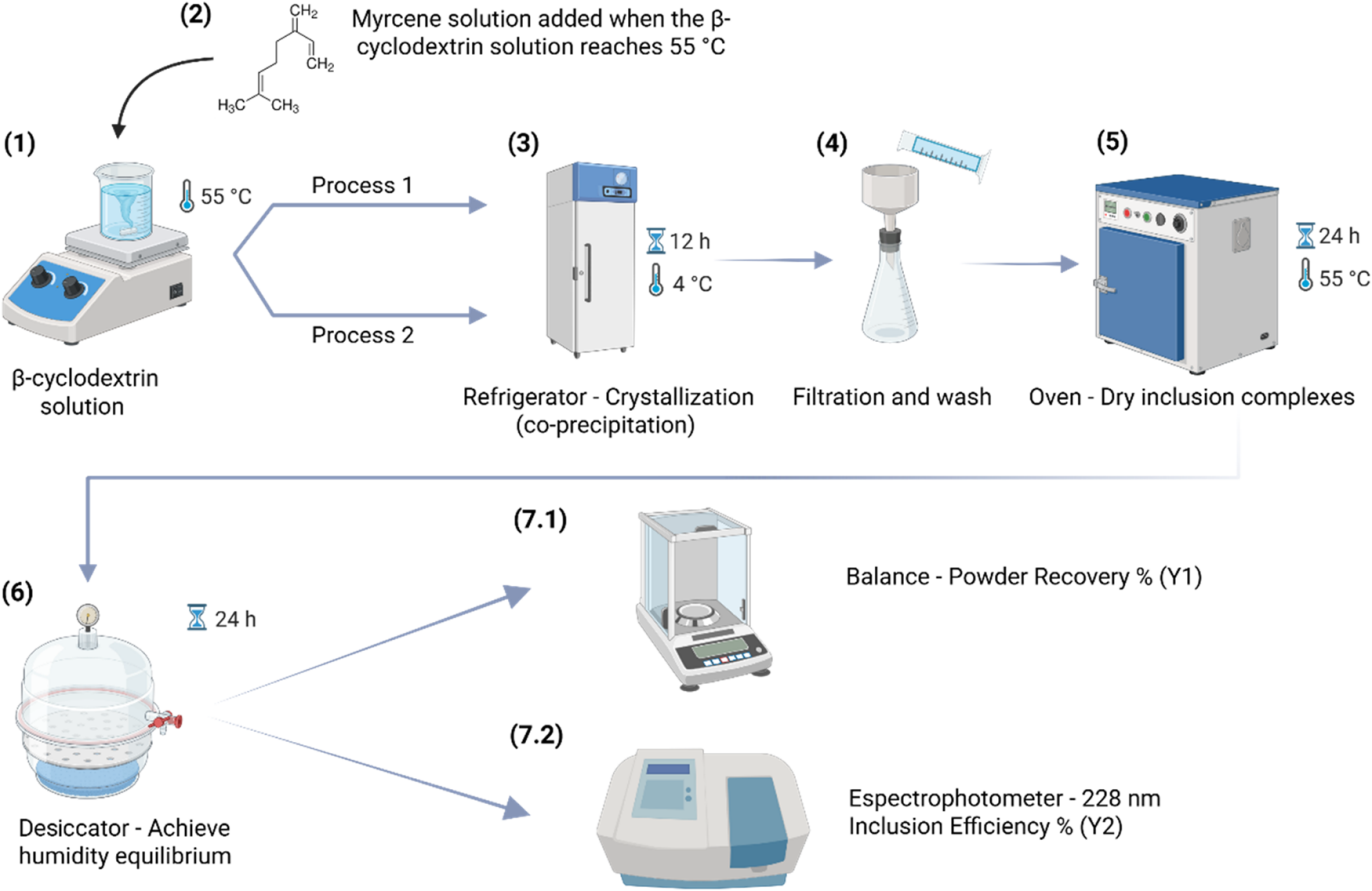
Inclusion complex (IC) obtained by the coprecipitation method. (1) Ethanol:water solutions containing β-CD; (2) myrcene solution added when (1) reaches 55 °C; (3) refrigeration overnight; (4) filtration and washing to remove myrcene not complexed; (5) oven for 24 h to remove water; (6) desiccator for 24 h for humidity equilibrium, and (7.1), (7.2) to evaluate the formed complexes. Source: *Created in BioRender. Duarte, J. (2026). Agreement number: PZ29NNBSAA.* https://BioRender.com/58s008g.

The reaction occurred under stirring, and the β-CD solutions were heated to 55 ± 2 °C. After reaching this temperature, the myrcene solution was slowly added in the proportions described above. In this step, after the addition of the myrcene solution, two processes were followed for the formation of the complexes: (i) maintaining heating at 55 °C for 30 min, and then continuous stirring for 4 h without heating; and (ii)­removing the heat immediately after the addition of the myrcene solution and stirring for 4 h.

Finally, the glass flask was refrigerated overnight at 4 °C, followed by vacuum filtration and washing with 10 mL of ethanol to remove the unencapsulated myrcene. They were then dried in an oven at 55 °C for 24 h and kept in a desiccator for another 24 h to reach the equilibrium moisture content. The complexes were weighed and preserved in glass vials at room temperature for later analysis and characterization.^19^

#### Optimization of Inclusion Complexes through Experimental Design

The complexes were optimized using a central response surface experiment composed of two independent variables (Table 6): myrcene:β-CD (solid/solid) and ethanol:water (liquid/liquid) at two range levels (2^2^) to determine their effects on the recovered powder (%) and myrcene retention efficiency (%). The results of processes 1 and 2 were evaluated separately using Minitab software through Response Surface Regression, specifically using the face-centered central composite design (FCCCD), with a *p*-value (<0.05) to determine the interference of these factors in the complexes formed.^19^

**Table 6:** Independent Variables and the Range of the Face-Centered Composite Central Response Surface Experiment

		**levels**		
**independent variables**	**unit**	**–1**	**0**	**+1**
X_1_: myrcene:β-CD ratio (w/w)	g/g	10:90	15:85	20:80
X_2_: ethanol:water ratio (v/v)	mL/mL	1:1	1:2	1:3

The comparison between processes 1 and 2 was performed using a *t*-test for two independent samples, considering a *p*-value of <0.05.

#### Powder Recovery (%) and Inclusion Efficiency (%)

The planning evaluation was based on the independent production factors of these complexes. Thus, at the end of the drying process, the samples were weighed (g), and the percentage of powder recovery was calculated following eq 4.^17^

4 $$powder\;recovery\;\left(\%\right)=\frac{\mathrm{Myrcene}:\beta ‐\mathrm{CD}\left(g\right)}{mMyrcene+m\beta ‐\mathrm{CD}\left(g\right)}\times 100\%$$

where Myrcene is the β-CD mass of the complex formed, weighed after drying; and *m*Myrcene + *m*β-CD is the initial mass used to produce the complexes.

To calculate the inclusion efficiency of myrcene in the complexes, the procedure described by Su (2019) was followed, with adaptations, for quantification by a UV–vis spectrophotometer, using the previously validated analytical method. 0.25 g of each sample was weighed, added to 10 mL of ethanol, kept under magnetic stirring for 2–3 min, and vacuum filtered with a microporous membrane to collect the filtrate. After UV quantification, the inclusion efficiency (%) was determined according to eq 5.^39^

5 $$inclusion\;efficiency\;\left(\%\right)=\frac{m\mathrm{Myrcene in}\;\beta ‐\mathrm{CD}\;\left(\mathrm{mg}\right)}{m\mathrm{Myrcene}\;\left(\mathrm{mg}\right)}\times 100$$

where *m*Myrcene in β-CD is the mass of myrcene present in the β-CD complexes, and *m*Myrcene is the initial amount of myrcene used to form the complexes.

### Characterization of the Inclusion Complex

#### Fourier Transform Infrared (FTIR) Spectroscopy

An IRPrestige-21 spectrometer (Shimadzu, Kyoto, Japan) was used for the analysis. The samples were homogenized with potassium bromide (KBr) at a 100:1 ratio, macerated in a porcelain mortar, and pressed into pellets for analysis. Myrcene, being a liquid substance, was spread across two sodium chloride windows and then analyzed in the same range as that of the other samples.^12,21^

The spectra of myrcene, β-CD, and physical mixtures in the proportions 10:90 and 15:85 (myrcene and cyclodextrin), as well as of the samples selected after the planning process (10:90 1:2, 10:90 1:3, and 15:85 1:3), were obtained. The experiment was conducted in the range of 4000 to 400 cm^–1^, with 4 cm^–1^ resolution in attenuated total reflectance (ATR) mode.

#### Thermogravimetric Analysis (TGA)

TGA 4000 (PerkinElmer, Waltham, Massachusetts, USA) was used in a temperature range of 30–600 °C, with a heating rate of 10 °C min^–1^ under a dynamic nitrogen atmosphere with a flow rate of 50 mL min^–1^ (Su et al., 2019). Samples were weighed in a porcelain crucible, with an average mass of 8.0 mg (±0.400 mg). The inclusion complexes selected after the design (10:90 1:2, 10:90 1:3, and 15:85 1:3), the β-cyclodextrin (β-CD) controls, and physical mixtures in the proportions 10:90 and 15:85 myrcene:β-cyclodextrin were analyzed.

#### Differential Scanning Calorimetry (DSC)

A DSC 4000 system (PerkinElmer, Waltham, Massachusetts, USA) was used in a temperature range of 25–350 °C, at a heating rate of 10 °C min^–1^ under a dynamic nitrogen atmosphere at a flow rate of 100 mL min^–1^.^29^ Samples were weighed in aluminum pans, with an average mass of 10 mg (±1.0 mg). The inclusion complexes selected after the design (10:90 1:2, 10:90 1:3, and 15:85 1:3), the β-cyclodextrin (β-CD) controls, and physical mixtures in the proportions 10:90 and 15:85 myrcene:β-cyclodextrin were analyzed.

#### X-ray Diffraction (XRD)

The analysis was performed using Smartlab SE equipment (Rigaku, Tokyo, Japan). Diffractograms were obtained at 20 mA and 40 kV using CuKa radiation (λ = 1.54 Å) at room temperature, with a scan range of 2–60° (scan step of 0.02 s^–1^). The analysis was conducted only for solid samples, inclusion complexes 10:90 1:2, 10:90 1:3, and 15:85 1:3, and β-cyclodextrin (control).^22^

#### Scanning Electron Microscopy (SEM)

The surface morphologies of β-CD and the inclusion complexes were examined using a JSM-IT500HR scanning electron microscope (Jeol, Boston, USA) at an operating voltage of 3.0 kV. Each sample was fixed in a metal support, coated with gold, and observed under 500x and 1000× magnifications.^17^

#### *In* Vitro Cytocompatibility in Keratinocyte Cell Lines

Biocompatibility was determined using HaCaT human keratinocytes (passages 19–22) as a representative skin cell line. For that, cells were cultured and maintained at 37 °C, 5% CO_2_, and 95% relative humidity in Dulbecco’s Modified Eagle’s Medium (DMEM) supplemented with fetal bovine serum (10%) (Gibco, Invitrogen, USA), penicillin (100 IU/mL), streptomycin (100 mg/mL), and amphotericin B (0.1%). 3 × 10^6^ cells per well were seeded on 96-well flat-bottom microplates and incubated for 24 h to allow attachment. Then, the medium was discarded, and the cells were exposed to myrcene for 24 h, β-cyclodextrin, and the inclusion complex 10:90 1:2. Samples were diluted in DMEM at a concentration range of 37.50–0.15 mg/mL for β-cyclodextrin and the inclusion complex. For free myrcene, inclusion concentrations equivalent to theoretical 100% were considered, following the the amount inside the complex (3,409.09 – 13.31 μg/mL), and kept in contact for 24 h at 37 °C. A positive control with 5 μL of stock SDS 10% was used. Cell viability was determined using the MTT reduction assay, which measures mitochondrial succinate dehydrogenase activity. For this, cells were exposed and incubated with 0.5 mg/mL MTT solution (100 μL/well; dissolved in the nutritional medium) for 1 h at 37 °C. After this time, the medium was removed, and 200 μL/well of DMSO was used to dissolve the formazan crystals.^31^ Absorbance was measured at 570 nm using a Synergy H1 microplate reader (Biotek, Vermont, USA), and cell viability was calculated using eq 6:

6 $$cell\;viability\;\left(\%\right)=\frac{Abs\;Sample-Abs\;Blank}{Abs\;Control-Abs\;Blank}\times 100$$

where Abs Sample is the absorbance measured from the formulation-exposed cells, Abs Control is the absorbance measured from the nonexposed cells, and Abs Blank is the base absorbance of the plate. The reported results were obtained as the average of three independent assays (*n* = 3).

#### Statistical Analysis

GraphPad Prism (version 8.5, San Diego, CA, USA) was used for statistical analysis of the data (from the MTT) for one-way ANOVA. Data are presented as mean values and standard errors (SEMs). *P*-values of <0.05 were considered significant.

## Supplementary Material



## Data Availability

All data supporting the findings of this study are included in the manuscript and accompanying Supporting Information. No additional data sets are required to support the conclusions of this work. Requests for further information regarding the data may be directed to Prof. Dr. Marlus Chorilli.

## Notes

Inclusion complex Process 1 and 2 results. Regression model coefficients and statistical parameters obtained by the *t-*test in Processes 1 and 2. Regression model coefficients and statistical parameters obtained by the *t-*test in Process 2 (PDF)
