# Supplementary material for: Preparation, Characterization, and Keratinocyte Cell Viability of a β‑Cyclodextrin–Myrcene Complex Intended for Skin Application
Source: ACS Omega. 2026 Jun 15;11(25):37454–66. doi: 10.1021/acsomega.6c01078 (PMC13325143; doi:10.1021/acsomega.6c01078)
Supplement: Supplementary file 1 [file ao6c01078_si_001.pdf]

## Supplementary Material

# Preparation, characterization and keratinocytes cell viability of a β-Cyclodextrin–Myrcene complex intended for skin application

Felipe Mota Tashiro[a], Jonatas Lobato Duarte[a], Miquel Martínez-Navarrete[b], Antonio José Guillot[b], Ana Melero[b], Marlus Chorilli[a]*

[a]Department of Drugs and Medicines, School of Pharmaceutical Sciences of Araraquara, São Paulo State University, São Paulo, Brazil; [b]Department of Pharmacy and Pharmaceutical Technology and Parasitology, University of Valencia, Valencia, Spain.

*Corresponding author.

**E-mail:** Felipe Mota Tashiro: felipe.tashiro@unesp.br, Jonatas Lobato Duarte: jl.duarte@unesp.br, Miquel Martínez-Navarrete: miquel.martinez-navarrete@uv.es, Antonio José Guillot: antonio.guillot@uv.es, Ana Melero: ana.melero@uv.es, Marlus Chorilli: marlus.chorilli@unesp.br.

**Table S1.** Process 1 - Heating for 30 minutes after adding myrcene.

| Sample | $X_1$ : Myrcene/ β-CD (m/m) | $X_2$ : Ethanol:water (v/v) | Initial mass (g) | Powder recovery (g) | $Y_1$ : Powder Recovery (%) | $Y_2$ : Inclusion Efficiency (%) |
|---|---|---|---|---|---|---|
| 1 | 10:90 | 1:1 | 1.1 | 0.7072 | 64.29 | 12.99 |
| 2 | 10:90 | 1:2 | 1.1 | 0.7450 | 67.73 | 12.66 |
| **3** | **10:90** | **1:3** | **1.1** | **0.7212** | **65.56** | **32.82** |
| 4 | 15:85 | 1:1 | 1.15 | 0.8649 | 75.21 | 9.84 |
| 5 | 15:85 | 1:2 | 1.15 | 0.8674 | 75.43 | 4.99 |
| **6** | **15:85** | **1:3** | **1.15** | **0.8373** | **72.81** | **19.26** |
| 7 | 20:80 | 1:1 | 1.2 | 0.8638 | 71.98 | 7.10 |
| 8 | 20:80 | 1:2 | 1.2 | 0.8821 | 73.51 | 3.50 |
| **9** | **20:80** | **1:3** | **1.2** | **0.8864** | **73.87** | **22.25** |

**Table S2.** Process 2 - No heating for 30 minutes after adding myrcene.

| Sample | $X_1$ : Myrcene/ β-CD (m/m) | $X_2$ : Ethanol:water (v/v) | Initial mass (g) | Powder recovery (g) | $Y_1$ : Powder Recovery (%) | $Y_2$ : Inclusion Efficiency (%) |
|---|---|---|---|---|---|---|
| 10 | 10:90 | 1:1 | 1.1 | 0.8291 | 75.37 | 39.06 |
| **11** | **10:90** | **1:2** | **1.1** | **0.84657** | **76.96** | **75.88** |
| **12** | **10:90** | **1:3** | **1.1** | **0.8466** | **76.96** | **66.66** |
| 13 | 15:85 | 1:1 | 1.15 | 0.8534 | 74.21 | 20.58 |
| 14 | 15:85 | 1:2 | 1.15 | 0.8541 | 74.27 | 42.01 |

| | | | | | | |
|---|---|---|---|---|---|---|
| **15** | **15:85** | **1:3** | **1.15** | **0.88791** | **77.21** | **53.13** |
| 16 | 20:80 | 1:1 | 1.2 | 0.9057 | 75.48 | 44.18 |
| 17 | 20:80 | 1:2 | 1.2 | 0.88197 | 73.50 | 33.66 |
| 18 | 20:80 | 1:3 | 1.2 | 0.8864 | 73.87 | 22.25 |

**Table S3.** Regression model coefficients and statistical parameters obtained by the t-test in Process 1.

| Parameters | $Y_1$ - Powder Recovery (%) | | $Y_2$ - Inclusion Efficiency (%) | |
|---|---|---|---|---|
| | **Estimated Coefficient** | **p-value** | **Estimated Coefficient** | **p-value** |
| Constant | 75.55 | <0.001 | 4.48 | 0.117 |
| $X_1$ | 3,629 | 0.010 | -4.27 | 0.032 |
| $X_2$ | 0.126 | 0.852 | 7.40 | 0.007 |
| $X_1^2$ | -4.99 | 0.019 | 3.86 | 0.141 |
| $X_2^2$ | -1.60 | 0.234 | 10.33 | 0.013 |
| $X_1.X_2$ | 0.153 | 0.854 | -1.17 | 0.457 |

Abbreviations: $\mathbf{Y_1}$: myrcene/β-CD ratio; $\mathbf{Y_2}$ **: ethanol** :water ratio.

**Table S4.** Regression model coefficients and statistical parameters obtained by the t-test in Process 2.

| Parameters | Y1 – Powder Recovery (%) | | Y2 - Inclusion Efficiency (%) | |
|---|---|---|---|---|
| | **Estimated Coefficient** | **p-value** | **Estimated Coefficient** | **p-value** |
| Constant | 75.10 | >0.001 | 44.17 | 0.018 |
| $X_1$ | -1,490 | 0.108 | -12.43 | 0.092 |
| $X_2$ | 0.084 | 0.906 | 7.53 | 0.236 |
| $X_1^2$ | -0.29 | 0.817 | 9.53 | 0.359 |
| $X_2^2$ | 0.19 | 0.876 | -8.39 | 0.412 |
| $X_1.X_2$ | -1,420 | 0.175 | -10.65 | 0.186 |

Abbreviations: $\mathbf{Y_1}$: myrcene/β-CD ratio; $\mathbf{Y_2}$**:** $_{\mathbf{ethanol}}$ :water ratio.
